# Quality of Artemisinin-based Combination Therapy for malaria found in Ghanaian markets and public health implications of their use

**DOI:** 10.1186/s40360-016-0089-2

**Published:** 2016-10-28

**Authors:** Mathilda Tivura, Isaac Asante, Albert van Wyk, Stephaney Gyaase, Naiela Malik, Emmanuel Mahama, Dana M. Hostetler, Facundo M. Fernandez, Kwaku Poku Asante, Harparkash Kaur, Seth Owusu-Agyei

**Affiliations:** 1 Kintampo Health Research Centre, Ghana Health Service/Ministry of Health, P O Box 200, Kintampo, Ghana; 2 London School of Hygiene and Tropical Medicine, London, WC1E 7HT UK; 3 Georgia Institute of Technology, School of Chemistry and Biochemistry, Atlanta, GA USA

**Keywords:** Ghana, Malaria, Artemisinin-based combinations, Counterfeit medicines, Falsified medicines, Substandard medicines, Morbidity, Mortality

## Abstract

**Background:**

Ghana changed their antimalarial drug policy from monotherapies to Artemisinin-based Combination Therapies in 2004 in order to provide more efficacious medicines for treatment of malaria. The policy change can be eroded if poor quality Artemisinin-based Combination Therapies are allowed to remain on the Ghanaian market unchecked by regulatory bodies and law enforcement agencies. The presence and prevalence of substandard and counterfeit Artemisinin-based Combination Therapies need to be determined on open markets in Ghana; a review of the current policy; identifying any gaps and making recommendations on actions to be taken in addressing gaps identified are essential as the data provided and recommendations made will help in ensuring effective control of malaria in Ghana.

**Methods:**

A field survey of antimalarial drugs was conducted in the central part of Ghana. The amount of active pharmaceutical ingredient in each Artemisinin-based Combination Therapy sample identified in the survey was measured using high performance liquid chromatographic analyses. Active pharmaceutical ingredient within the range of 85–115 % was considered as standard and active pharmaceutical ingredient results out of the range were considered as substandard. All samples were screened to confirm stated active pharmaceutical ingredient presence using mass spectrometry.

**Results:**

A total of 256 Artemisinin-based Combination Therapies were purchased from known medicine outlets, including market stalls, hospitals/clinics, pharmacies, drug stores. Artemether lumefantrine (52.5 %) and artesunate amodiaquine (43.2 %) were the predominant Artemisinin-based Combination Therapies purchased. Of the 256 Artemisinin-based Combination Therapies purchased, 254 were tested, excluding two samples of Artesunate-SP. About 35 % of Artemisinin-based Combination Therapies were found to be substandard. Nine percent of Artemisinin-based Combination Therapies purchased were past their expiry date; no counterfeit (falsified) medicine samples were detected by either high performance liquid chromatographic or mass spectrometry.

**Conclusion:**

A high proportion of Artemisinin-based Combination Therapies sold in central Ghana were found to be substandard. Manufacturing of medicines that do not adhere to good manufacturing practices may have contributed to the poor quality of the Artemisinin-based Combination Therapies procured. A strict law enforcement and quality monitoring systems is recommended to ensure effective malaria case management as part of malaria control.

## Background

Though the therapeutic effectiveness of medicines to cure common tropical diseases in Africa is a poverty remedial measure, the integrity of these medicines is critical in successfully combating the diseases they target [1, 2]. Counterfeit and substandard medicines pose a stumbling block to public health delivery systems for malaria in endemic countries, [3–5]. Meta-analysis of existing data reported roughly 30 % poor quality medicines in South East Asia and sub-Saharan Africa [2].

Malaria and microbial infections are the leading causes of morbidity and mortality in Ghana [6, 7]. Malaria, alone, accounts for about 40 % of outpatient visits in the country and has significantly contributed to morbidity in all age groups [8, 9].

Concerns have recently been raised in Ghana and several other African countries about resistance to prescribed medicines for treatment of diseases [10]. Among reasons for the loss of medicines efficacy are resistance of pathogens to medicines that were previously effective; non-compliance of patients to treatment and dosage regimes and proliferation of counterfeit and/or substandard medicines [4, 11].

Since the introduction of Artemisinin-based Combination Therapy (ACT) in Ghana in 2004, there has been proliferation of different brands of ACTs on the Ghanaian market. It is perceived that some of the ACTs could be either counterfeits or substandard [12]. Surveillance on poor quality medicines could potentially be insufficient in Ghana to address this challenge, thus putting the lives of many Ghanaians at risk [13–16]. The situation is being exacerbated by the culture of indiscriminate ‘over the counter sale’ of all categories of medicine in the country [16, 17].

In response to recent reports in the Ghanaian media [18] of poor quality ACTs, we purchased following the mystery shopping strategy, all types of ACTs available in predominantly rural communities in the middle part of Ghana, in order to determine their quality using chromatographic methods established at the London School of Hygiene & Tropical Medicine (LSHTM) and the Georgia Institute of Technology, Atlanta, USA [19].

## Methods

### Study area

A cross-sectional survey was carried out between January 2011 and June 2011 in the Kintampo Health Research Centre (KHRC) study area located in 8 neighbouring districts, made up of Kintampo North, Kintampo South, Nkoranza North, Nkoranza South, Techiman North, Techiman South, Tain and Wenchi in the central part of Ghana. These districts are predominantly rural with close to 80 % of the resident population living in rural communities. The tribal groups mainly consist of Bono and Mo origins with a significant part of residents having migrated from the northern parts of the country. The occupation is mainly subsistence farming of crops such as yam, cassava and maize [20]. Malaria is the disease of highest burden in the area. [21–23].

The health systems in these districts are largely basic and modelled so that they follow a uniform pattern used in district health systems in Ghana. The lowest level (referred to as level A) of health care is at the community level and manned by Community Health Nurses. The next level is Health Centre level (referred to as level B), where middle level health professionals (physician assistant, midwife, nurses, laboratory and dispensary technicians are in charge of the system. The district hospital is level C and employs senior-level health professional including physicians, anaesthetists, senior nurses/midwifes, pharmacists and laboratory technologists. Patients presenting at the lower level with any condition that cannot be managed are referred to the next level for management; with cases beyond district hospitals referred to regional hospitals or teaching hospitals. Health seeking by most community members needing treatment, however, does not always follow this conventional arrangement as home treatment of malaria using drugs purchased from licensed chemical sellers is the commonest health seeking behaviour [24–26].

The study area consisted of 237 medicine outlets (MO); these comprised 37 health facility dispensaries, eight pharmacies and 192 license chemical shops. Mobile vendors and some grocery shops, drinking bars and homes were other sources of medicine sales; these were mostly accessible to community members since they are located within the community. The quality of drugs are to be regulated by the Ghana Food and Drugs Authority in collaboration with the Pharmacy Council of Ghana and various security agencies (including police, Customs Excise & Preventive Services among others) are charged with ensuring that only quality medicines are available on the market.

### Medicine outlet selection

The Kintampo Health and Demographic Surveillance database, which has geolocations of all communities, MOs and health facilities, was used to create MO clusters. Cluster A included MOs within a 5-km radius from a district/municipal hospital, cluster B included MOs within 5–10 km radius from a district/municipal hospital and cluster C included MOs >10 km from a district/municipal hospital. Within each cluster, 10 MOs were randomly selected if the number of known MOs was less than forty and 20 MOs were randomly selected if the number of outlets was 40 or higher. All pharmacies were, however, purposively selected for medicines to be purchased due to their limited number [8] in the studied area. All mobile vendors and other outlets in the selected communities were included for medicine procurement. Medicines outlets were stratified by clusters as defined above and 141 of these outlets were randomly selected from a total of 237 medical outlets within the study.

A “mystery shopping” strategy was used to buy ACTs from the selected shops [19]. Trained mystery shoppers were made to visit each selected MO, present themselves as a client with malaria symptoms and engage the shop attendant in a discussion regarding what antimalarials were available for sale to clients. From the list communicated, the mystery shopper purchased some of the ACTs available in stock and then made a mental note of all other ACTs that were available and could be obtained from the MO. The information was then relayed to another mystery shopper who later visited the shop to purchase the other brands of ACTs available in the shop. The process of mystery shopping was repeated in all identified shops until samples of all brands of ACTs available in each selected shop were obtained. The trained shoppers carried through this process in such a way that there was never any suspicion by the sellers.

Mystery shoppers wrote down the description of the outlet on exiting the MO. ACTs purchased were transported to the Kintampo Health Research Centre Pharmacy on daily basis. All samples purchased were stored as per the manufacturer’s instructions by the study pharmacist.

The Regional Medical Store was visited to ascertain the state and storage conditions of ACTs that were in stock.

Samples were shipped within two months to the bioanalytical facility at the London School of Hygiene and Tropical Medicine (LSHTM) for analysis. The packaging on each sample was scanned electronically and/or photographed at LSHTM and a detailed description of the ACT was recorded on a Microsoft Excel spreadsheet. The description included brand name, stated active ingredients, dose form, date of purchase from MO, name of manufacturer, country of origin, batch number, expiry date, number of tablets per packet and retail price paid. The database created together with the scans of the packets and their contents was shared with the Ghana team.

### Chemical content analysis and classification of samples

Laboratory assessment of the quality of ACTs purchased in the central part of Ghana was carried out at the London School of Hygiene and Tropical Medicine (LSHTM) and the Georgia Institute of Technology (GT). Sample packaging and appearance were assessed, coded and recorded, as well as the weight, and dimensions of each sample. The amount of active pharmaceutical ingredient (API) in each sample was determined using high performance liquid chromatography (HPLC) and the presence of any compounds other than the stated APIs was screened by ambient mass spectrometry (MS) following published methods [19]. Formulations were analysed for the amount of each active pharmaceutical ingredient (API) present using high performance liquid chromatography (HPLC). Briefly each tablet was pulverised, dissolved in solvent depending on the stated API; artesunate (AS), artemether (AM), dihydroartemisinin (DHA), amodiaquine (AQ) and sulfadoxine/pyrimethamine (SP) were dissolved in methanol; samples containing lumefantrine (LUM) were dissolved in 10 % acetic acid in methanol, and piperaquine (PIP) in methanol/0.1 M HCl (1:1; v/v). Solvent extracts were sonicated followed by centrifuging, and the supernatant injected into the HPLC column to determine the amount of API present.

HPLC analysis was conducted using a Dionex Ultimate 3000 system (Thermofisher, Hemel Hempstead, UK) and separation achieved using a GENESIS AQ 4 μm column (150 × 4.6 mm, Grace Materials Technologies, Cranforth, UK). The mobile phase was a gradient of ammonium formate (10 mM, pH 2.7) and acetonitrile (v/v; 60:40 to 85:15 over 7.0 min). A photo-diode array unit (UV-PDA; DAD 3000) was set at 204 nm for the artemisinin derivatives (AS, AM, DHA), 360 nm for PIP, AQ and LUM. In all cases, the flow rate used was 1.0 ml/min. Calibration curves of each compound were generated by Thermofisher Scientific Dionex Chromeleon 7.2 chromatography data system (CDS) software using known amounts of the corresponding chemical standards. Reference standards of artemisinin, artesunate, artemether, dihydroartemisinin, amodiaquine dichlorodihydrate and pyrimethamine were purchased from Sigma Aldrich, UK. Lumefantrine was purchased from WHO, Switzerland. Results were expressed as a percentage of the stated amounts of API on the package.

Quality of ACT was assessed by comparing the amount of API detected with that stated on the packaging label and indicated as a percentage of the stated value.

We adopted a range between 85 % and 115 % of the stated API content for both compounds in the ACT to classify samples as being of acceptable quality. Medicines with less than 85 % or over 115 % of the stated API content of any of the partner compounds were therefore classified as substandard.

### Data management and analysis

The questionnaires completed by mystery shoppers as well as basic data collected by the pharmacist were double-entered, range and consistency checks and verification were carried out using Microsoft Access. The sampling was randomised and all data were cleaned and analyzed using STATA version 11 (Stata Corp., TX USA). Simple descriptive analysis such as proportions are used to summarize various variables including the prevalence of substandard drugs. Bar graphs were used to summarize some results such as sources of ACTs and country of their origin using Microsoft Excel.

## Results

ACTs were found in 107 out of the 141 selected MOs. The majority (81.3 %) of MOs visited were license Chemical Sellers Shops (Table 1).

**Table 1:** Types and availability of ACTs in the Medicine outlets surveyed

Drug Outlet	Total number of outlets visited stocking ACTs	% outlets stocking ACTs
License Chemical Shops	87	81.3
Pharmacy	7	6.5
Health Facility	7	6.5
Mobile vendor	2	1.9
Other^a^	4	3.8
Total	107	100.0

^a^Other comprised of drinking bars, grocery shops, homes and mark

Two hundred and fifty six ACT samples were purchased by the mystery shoppers. Approximately 9 % of the ACTs purchased had already expired. Artemether-lumefantrine (AL) and artesunate-amodiaquine (AA) were the leading ACTs in the market (Table 2). A few Licensed Chemical Sellers shops and pharmacy shops also had dihydroartemisinin piperaquine (DHAP) in stock. All AA samples purchased were coblistered.

**Table 2:** Types of ACTs purchased from the various medicine outlets (MO)

Type of ACT	Health Facilities	Licensed chemical shops	Pharmacy	Mobile vendor	Others	Total
	*n* (%)	*n* (%)	*n* (%)	*n* (%)	*n* (%)	*N* (%)
AL	4 (21.1)	101 (54.6)	25 (83.3)	3 (42.9)	1 (7.1)	4 (52.3)
AA	15 (78.9)	77 (41.4)	3 (10.0)	4 (57.1)	12 (85.7)	111 (43.4)
DHAP	0 (0.0)	7 (3.8)	2 (6.7)	0 (0.0)	0 (0.0)	9 (3.5)
ASP	0 (0.0)	1 (0.5)	0 (0.0)	0 (0.0)	1 (7.1)	2 (0.8)
TOTAL	19 (100)	186 (100)	30 (100)	7 (100)	14 (100)	256 (100)

Others: include ACTs purchased from drinking spots and homes

*AL* Artemether-Lumefantrine, *AA* Artesunate-Amodiaquine, *DHAP* DihydroartemisininPiperaquine, *ASP* Artesunate-Sulfadoxine-Pyrimethamine

It was found that 35.4 % (90/254) of the ACTs were substandard and failed to comply with the required amount of APIs (Table 3). This means that only 64.6 % (164/254) were in the recommended API range (Table 3). No grossly counterfeit (falsified) medicine samples were detected by either HPLC or MS. Only two samples, artesunate-SP, were found and purchased at the sampled MOs, but these were not tested for logistics reasons.

**Table 3:** Number of ACTs complying with API quality tests using High Performance Liquid Chromatography (HPLC)

HPLC results	Number	Percent
Acceptable for both AD and PM	164	64.6
Acceptable for AD and Substandard for PM	54	21.3
Acceptable for PM and Substandard for AD	23	9.0
Substandard for both AD and PM	13	5.1
Total	254	100.0

Note: AD stands for Artemisinin Derivative, PM stands for Partner medicines

In order to document the contribution of ACT samples that did not comply with the amount of stated API, the component of each brand of ACT was quantified. For samples stated to contain artesunate, 22.5 % (25/111) had the artemisinin components at levels lower than the stated API content. Also 4.5 % (6/134) of artemether samples tested had artemether components at levels lower than the stated API content (Table 4). On the contrary, none (0/111) and 1.5 %(2/134) of samples tested had artemisinin and artemether components at levels greater than the required API content, respectively (Table 4).

**Table 4:** HPLC Analysis of Artemisinin derivative (AD) of ACT

Artemisinin Derivative	Below 85 %	Within 85–115 %	Above 115 %	Total
	*n* (%)	*n* (%)	*n* (%)	*N* = 254
Artesunate^a^ n (%)	25 (22.5)	86 (77.5)	0 (0.0)	111 (100.0)
Median (IQR)	80.0 (77.2–82.7)	92.9 (89.5–98.6)	0	91.5 (86.0–95.8)
Artemether^b^ n (%)	6 (4.5)	126 (94.0)	2 (1.5)	134 (100.0)
Median (IQR)	76.6 (66.8–77.5)	95.9 (92.1–100.4)	116.1 (115.1–117.0)	95.6 (91.5–100.5)
Dihydroartemisin^c^ n (%)	0 (0.0)	6 (66.7)	3 (33.3)	9 (100.0)
Median (IQR)	0	105.1 (89.2–107.3)	132.4 (120.3–143.2)	107.3 (103.4–120.3)
Combined n (%)	31 (12.2)	218 (85.0)	5 (2.0)	254 (100.0)
Median (IQR)	78.7 (76.7–82.5)	94.6 (90.9–99.9)	120.3 (117.0–132.4)	93.6 (89.4–99.7)

Note: ^a, b, c^were derived from Artesunate Amodiaquine. Artemether Lumefantrine and Dihydroartemisinin Piperaquine respectively

Analysis of other medicines, such as amodiaquine, used in combination with artemisinin derivatives showed that 25.2 % (28/111) of samples had contents lower than that stated. Similarly, 3.0 % (4/134) of samples tested had lower than required lumefantrine content (Table 5). Higher than acceptable amodiaquine and lumefantrine content was found in 13.8 % and 30.1 % of the tested ACT samples, respectively (Table 5).

**Table 5:** HPLC results for Partner medicines (PM)

Partner Medicines	Below 85 %	Within 85–115 %	Above 115 %	Total *N* = 254
Amodiaquine^a^ n (%)	28 (25.2)	64 (57.7)	19 (17.1)	111 (100.0)
Median (IQR)	80.6 (72.6–82.9)	92.7 (88.3–97.2)	118.8 (117.2–121.4)	92.0 (84.9–101.7)
Lumefantrine^b^ n (%)	4 (3.0)	120 (89.6)	10 (7.5)	134 (100.0)
Median (IQR)	83.3 (77.4–84.4)	99.4 (93.2–105.0)	117.6 (116.2–118.5)	100.3 (93.1–107.1)
Piperaquine^c^ n (%)	0 (0.0)	3 (33.3)	6 (66.7)	9 (100.0)
Median (IQR)	0	112.1 (111.5–114.7)	121.1 (117.5–123.8)	117.5 (114.7–121.7)
Combined n (%)	32 (12.6)	187 (73.6)	35 (13.8)	254 (100.0)
Median (IQR)	81.4 (72.8–83.0)	96.6 (91.6–103.4)	118.7 (117.2–120.7)	96.8 (88.9–108.4)

Note: ^a, b, c^were derived from Artesunate Amodiaquine. Artemether Lumefantrine and Dihydroartemisinin Piperaquine respectively

**Fig. 1:**
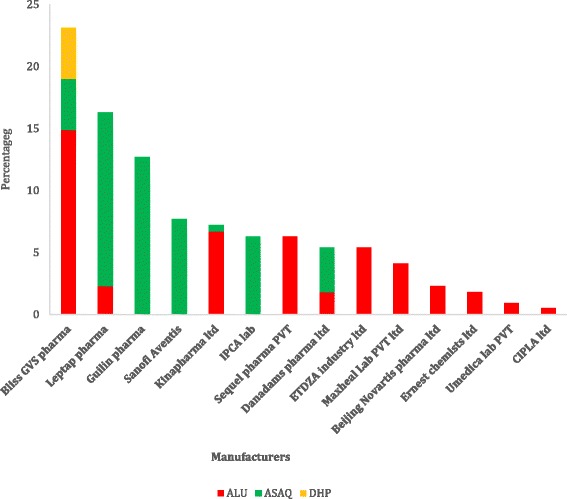
The percentages of ACTs made by each manufacturer in our market survey in central Ghana. In Fig. 1, is a bar chart, which illustrates the manufacturers of ACTs found on the market in central Ghana. The ACTs from each manufacturer were expressed as percentages. The x-axis represents the manufacturing companies whose ACT products were found in the market in central Ghana. The y-axis illustrates the percentage proportion of each ACT produced by each manufacturer, among the identified manufacturing companies

About 52 % of ACT market share is taken up by three pharmaceutical companies (Fig. 1).

**Fig. 2:**
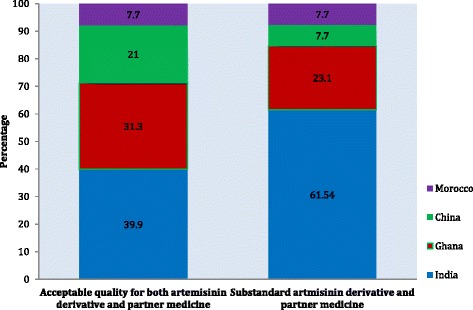
Percentage of the total number of substandard ACTs originating from each country of production. Fig. 2 is an illustration of the HPLC results of ACTs by countries of manufacture. The x-axis represents the results whilst the y-axis indicates the percentage proportion for each country. The portions of the graph coloured blue represent Indian manufacturing companies. The portions of the graph coloured red represent Ghanaian manufacturing companies. The portions of the graph coloured green represent Chinese manufacturing companies. The portions of the graph coloured violet represent Moroccan manufacturing companies

ACTs labelled with India as the country of origin comprised the highest proportion (61.54 %) of substandard ACTs (Fig. 2). This was followed by ACTs produced in Ghana (23.1 %), China (7.7 %) and Morocco (7.69 %).

## Discussion

Malaria remains the most frequent reason for seeking healthcare in Ghana and registers the highest level of mortality [27]. In 2004, Ghana changed malaria policy from the use of monotherapies to ACT. This study was carried out in response to increasing anecdotal reports of substandard and counterfeit ACTs in the Ghanaian market since the start of implementation of policy changes in 2005 [28].

The prevalence of substandard ACTs was relatively high; 35.4 % of all ACTs purchased were substandard and contained less than the stated amount of API. Specifically, the majority of substandard artesunate-amodiaquine ACTs had lower than expected levels of artesunate, the more expensive API. Reasons that could have led to this high prevalence of substandard ACTs include poor compliance with Good Manufacturing Practices (GMP) and/or poor storage conditions along the supply chain. The relative contribution of these two factors could not be ascertained easily and is beyond the scope of this study.

Artemether-lumefantrine (AL) and artesunate-amodiaquine (AA) were the commonest ACTs on the market and are the ones recommended by the Ghana National Malaria Control Programme (GNMCP); however the investigators were unable to ascertain if all the brands and batches purchased in this survey had been registered with the Ghana Food and Drugs Authority (GFDA). A set of all samples of ACTs purchased have, however, been shared with GFDA. It is also possible that some of these medicines were not officially approved.

ACTs found in the Ghanaian markets are not unique to Ghana but can also be found in other malaria endemic countries. Levels of poor quality ACTs comparable to those reported in this manuscript have also been found in Asia and other parts of Africa, as wide networks and distribution channels already exist; it therefore means that issues regarding poor quality medicines are global in nature and require serious attention. Studies carried out in Southeast Asia (e.g. Cambodia, Laos, Myanmar, Thailand, Vietnam, China and Indonesia) and Africa (e.g. Senegal, Madagascar, Uganda, Burkina Faso, Nigeria and Ghana) [29–35] confirm high amounts of substandard antimalarials in circulation. Adherence to rules and regulations regarding importation and in-country manufacture of ACTs has led to minimal levels of substandard and virtually non-existence of counterfeit ACTs in countries such as Ethiopia and Rwanda [32]. The main outstanding challenge in Ethiopia has been to ensure that all medicines are registered to rule out any vulnerability towards penetration of poor quality products [32].

Recent research on ACTs has shown that products from WHO-approved manufacturers can be reported to be up to five times less likely to fail basic quality tests [36]. It is therefore important that a thorough audit be carried out on all manufacturers who are without WHO approval. A strong regulatory system is key in preventing substandard drugs entering the consumer market. In Rwanda there is an existing mandatory system for all manufacturers to provide a current WHO-approved certificate of Good Manufacturing Practice before the Ministry of Health can award contracts to those qualified to supply medicines. Rwanda uses only WHO-approved certified manufacturers [36], and this has helped in ensuring high quality standard ACTs prevail in the markets. Other countries should emulate Rwanda’s tight processes where its bureau of standards verifies, documents and together with their customs services department and ministry of health, inspects all medications imported into the country. Where poor quality medicines are found, medicine regulatory authorities partner with Rwandan police force and Interpol to target the organized criminals responsible, whenever possible. Thus, Rwanda’s drug quality control strategy depends not only on its health system, but also on its law enforcement and justice systems [37].

The sources of substandard ACTs found in the study area are global as substandard ACTs from all ACT manufacturers were observed. Substandard ACTs were identified from pharmaceutical companies in both Africa and Asia with notable countries being India, Ghana, China and Morocco (Fig. 2). A concerted effort by Ghanaian authorities will be required in this regard to drive the reduction and eventual extermination of both production and importation of poor quality ACTs.

Co-blistered artesunate-amodiaquine was the commonest ACT formulation found in the Ghanaian market. To improve on compliance, it should be the highest priority of pharmaceutical companies to replace co-blistered formulations with co-formulated ones, as patients can be selective towards the artemisinin component at the expense of the amodiaquine one, thereby increasing the likelihood of developing Plasmodium parasite strains resistant to artemisinins.[28, 38, 39].

Regional medical stores, government dispensaries and other pharmacies located in the study area were not compliant to the Ghana national drug poilcy requirements [40], as there was no thorough adherence to best practices involving the equipment, design and regular maintenance of medicine storage facilities. Records of GFDA monitoring visits in most of the facilities sampled could not be readily found. Also, some facilities had expired medicines in stock that should have been disposed of in accordance with national guidelines [40].

Proliferation of substandard ACTs into the country was observed in this survey.

When the policy and programme response against counterfeit and substandard ACTs was reviewed, it was noticed that documentations on monitoring and surveillance on medicines supply chains existed. These included procurement, storage conditions and distribution networks. The role of pharmacists, licensed chemical sellers, consumers of the medicines as well as public awareness programmes were captured in the Ghana drug and antimalarials policies as referenced in the Republic of Ghana National Drug Policy [41] as well as the Anti-malaria Drug Policy for Ghana [42].

The main reasons attributed to proliferation of substandard ACTs are lack of enforcement of drug policies by the national regulatory body and law enforcement institutions in Ghana. Resource and capacity constraints contribute to this less-than-ideal situation [43, 44]. The national borders remain porous, making it easy for importation of poor quality medicines into the country, thereby risking the lives of patients. The roles of the GFDA, Pharmacy Council of Ghana (PCG) and other authorities (including Ghana customs, Ghana police etc.) need to be more clearly defined. These agencies should be empowered to perform their roles without fear or favour.

It is promising that discussions are currently on-going among WHO-Member States about quality, safety and efficacy of antimalarials [45].

The findings of our study may seem re-assuring as no grossly counterfeit ACTs were detected. Nevertheless the high prevalence of substandard ACTs is highly concerning and should be treated with the highest public health priority. Most of the patients receiving these poor quality ACTs live in area without documented resistance to ACTs, but resistance could develop in future. Furthermore, these patients may not be appropriately treated for their illness when the need arises. Routine drug quality monitoring strategies should be put in place to gain a better understanding of the extent of the problem and changes that may occur over time, so that the malaria control programme can respond with any control measures needed. Proliferation of poor quality ACTs in malaria endemic countries has the potential for favouring selection of resistant malaria parasite species that could otherwise have been treated effectively leading to progressive loss of confidence in modern medicines by the Ghanaian populace,

### Limitations

The data used in this study was from 8 out of the current 216 districts in Ghana. There is the possibility that actions and recommendations made based on the findings of this survey may not be able to eradicate the practice of importation into or manufacture of poor quality ACTs in Ghana. This survey was, however, carried out in typical districts in Ghana and therefore the findings are deemed to be representative of the national situation.

The non-disclosure of intent of the research team to the sellers at the MOs and suppliers of the ACTs may raise some concerns about the design of this study; but recommendations of the ethics review committee on the use of “mystery shoppers” were followed. Though disclosure of the full intent of the shoppers could have been used, this may have triggered unwillingness of medicine sellers to cooperate with our study team, as well as put them in harm’s way. Our risk-benefit balance therefore leaned towards non-disclosure of the identities of the “mystery shoppers”.

### Recommendations

To effectively combat proliferation of poor quality medicines and ensure only high quality medications exist on the Ghanaian market:

- □ There should be first and foremost, mechanisms in place for effective inter-country collaboration, since much of the trade in poor quality medicines occurs across national borders. There is the need for the regulatory authorities in Ghana to map out all entry points for imported medicines so that surveillance on medicines imports can be more effectively deployed at all entry points; ensuring that medicines entering Ghana are registered and have passed appropriate quality control testing. These measures will also require that regulatory agencies are provided the much needed personnel and equipment to work more effectively. Additionally, advertisement and promotion of medicines should be regulated as well.
- □ The Ghana Food and Drugs Authority (GFDA) should maintain a list of medicine manufacturers to help it decide how monitoring and surveillance of ACT quality could be tackled effectively. The entire medical supply chain needs tightening to eliminate any loopholes that may exist. Random surveys through the open market should be regularly enforced to quarantine all medicines that have not yet gone through GFDA registration.
- □ Legislation, including how medicines will be tested for quality, should be updated and streamlined to keep pace with the scale of the drug quality problem and to provide a dissuasive deterrent to criminal activity. The global health community and national governments will need to engage with consumers so that patients understand the importance of only buying medicines through regulated outlets. Health care providers such as physicians, nurses and pharmacist are well positioned to help governments in this difficult fight by reporting any suspicious ACTs that they come across in their work. They can also educate their patients on how to identify poor quality medicines by using visual aids. Giving incentives to individuals who provide critical information leading to the conviction of the culprits of this crime may also be needed. Great emphasis on the overall strategies suggested here should be put in rural areas where current control and monitoring of the medicine quality is generally more challenging.
- □ There should be regular testing on randomly-selected antimalarials found in known outlets.
- □ It is recommended that co-blistered ACTs be banned, with only co-formulated ACTs remaining in the Ghanaian market so as to improve patient adherence.

## Conclusions

No counterfeit ACTs were found when purchases were made in the studied area, however, substandard ACTs were very common. It was found that 35.4 % of the ACT medicines sampled API content. About 9 % of the ACTs for sale to community members at MOs had already expired.

Though this work was carried out in eight typical districts in Ghana, it is not expected that the results will be different in other geographical areas in the country. It can be inferred that there are major public health implications for Ghana and other malaria endemic countries with such poor quality medicines flooding the health systems. While ACTs are still highly efficacious for malaria treatment in most cases, several factors detract from their effectiveness. These not only include drug quality, but also access to ACTs, prescriber compliance, and patient adherence. In areas with high percentages of substandard or expired ACTs, there is worry that a fraction of those who have access to ACTs for treatment will not have their malaria treated appropriately, leading to continuing high levels of morbidity and mortality, loss of days at work, excessive pressure and loss of confidence on health systems, resorting to traditional remedy. All organizations and individuals championing the message of controlling, eliminating and eradicating malaria eventually need to be informed regarding the prevalence of poor quality medicines.

## Abbreviations

AA: Artesunate-amodiaquine
ACT: Artemisinin-based combination therapy
AD: Artemisinin derivative
AL: Artmether-lumefantrine
AM: Artemether
API: Active pharmaceutical ingredient
AQ: Amodiaquine
AS: Artesunate
CDS: Chromatography data system
DHA: Dihydroartemisinin
DHAP: Dihydroartemisinin piperaquine
GFDA: Ghana Food and Drug Authority
GMP: Good manufacturing practice
GNMCP: Ghana National Malaria Control Programme
GT: Georgia Institute of Technology
HPLC: High performance liquid chromatography
IQR: Inter quartile range
KHRC: Kintampo Health Research Centre
LSHTM: London School of Hygiene and Tropical Medicine
LUM: Lumefantrine
MO: Medicine outlets
MS: Mass spectrometry
PCG: Pharmacy Council of Ghana
PIP: Piperaquine
PM: Partner Medicine
SP: Sulfadoxine/pyrimethamine
UK: United Kingdom
USA: United State of America
WHO: World Health Organisation

## References

[1] Newton PN, McGready R, Fernandez F, Green MD, Sunjio M, Bruneton C (2006). Manslaughter by Fake Artesunate in Asia- Will Africa Be Next?. PLoS Med.

[2] Nayyar GM, Breman JG, Newton PN, Herrington J (2012). Poor-quality antimalarial drugs in Southeast Asia and sub-Saharan Africa. Lancet Infect Dis.

[3] World Health Organization. Country Cooperation Strategies At a Glance. 2012a, http://www.who.int/countryfocus/cooperation_strategy/en Accessed 20 Jul 2013

[4] Dondorp AM, Newton PN, Mayxay M, Van Damme W, Smithuis FM, Yeung S (2004). Fake antimalarials in Southeast Asia are a major impediment to malaria control: multinational cross-sectional survey on the prevalence of fake antimalarials. Trop Med Int Health.

[5] Newton PN, White NJ, Rozendaal JA, Green MD (2002). Murder by fake drugs: time for international action. BMJ.

[6] USAID. 2013. Country Profile- President’s Malarial Initiative Ghana, viewed 20 July 2013, https://www.pmi.gov/docs/default-source/default-document-library/country-profiles/ghana_profile.pdf?sfvrsn=22.

[7] Newman MJ, Frimpong E, Donkor ES, Opintan JA, Asamoah-Adu A. Resistance to antimicrobial drugs in Ghana. Infection and drug resistance, 2011;4:215-20. 10.2147/IDR.S21769 PMC3259688 22259250

[8] World Health Organization. 2012b, Update on artemisinin resistance April 2012 http://www.who.int/malaria/publications/atoz/arupdate042012.pdf. Accessed 29 July 2013.

[9] GNMCP 2011. Ghana National Malaria Control Programme. Final report, 2011. http://www.ghanahealthservice.org/download/ghana_malaria_programme_review_final_report_june_2013.pdf. Accessed 29 July 2013.

[10] World Health Organization. Survey of the quality of selected antimalarial medicines circulating in six countries of sub-Saharan Africa. World Health Organization; 2011a. pp 118

[11] Newton PN, Green MD, Mildenhall DC, Plancon A, Nettey H, Nyadong L, et al. Poor quality vital anti-malarials in Africa – an urgent neglected public health priority. Malaria J. 2011. 10;352: doi:10.1186/1475-2875-10-352 10.1186/1475-2875-10-352 PMC3262771 22152094

[12] Klein EY, Lewis IA, Jung C, Llinás M and Levin SA. Relationship between treatment-seeking behaviour and artemisinin drug quality in Ghana. Malaria J. 2012. 11;110:doi: 10.1186/1475-2875-11-110 10.1186/1475-2875-11-110 PMC3339389 22482747

[13] Forzley M. Combating Counterfeit Drugs: Concept Paper for an International Framework Convention And Related Strategies. World Health Organization Department of Essential Medicines and Policy. 2004

[14] Gaudiano MC, Di Maggio A, Cocchieri E, Antoniella E, Bertocchi P, AlimontiS et al. Medicines informal market in Congo, Burundi and Angola: Counterfeit and sub-standard antimalarials. Malaria J. 2007. 6;22:doi:10.1186/1475-2875-6-22 10.1186/1475-2875-6-22 PMC1810297 17316432

[15] Weigmann K (2013). Elixirs of death. EMBO Rep.

[16] Ofori-Kwakye KT, Asantewaa Y, Gaye O (2008). ‘Quality of Artesunate Tablet Sold in Kumasi. Ghana’ Trop J Pharmaceutical Res.

[17] Asamoah D, Abor P, Opare M (2011). An examination of pharmaceutical supply chain for artemisinin-based combination therapies in Ghana’. Manag Res Rev.

[18] Ghanaweb2013:http://www.ghanaweb.com/GhanaHomePage/health/artikel.php?ID=273570. Accessed 3 Aug 2013.

[19] Kaur H, Elizabeth LA, Ibrahim M, Zoe H, Ogochukwu I, Mohamed E-S (2015). Quality of Artemisinin-Based Combination Formulations for Malaria Treatment: Prevalence and risk factors for poor quality medicines in public facilities and private sector drug outlets in Enugu, Nigeria. PLoS ONE.

[20] Ghana Statistical Service 2013, 2012 Population & Housing Census National Analytical Report accessed 13 July 2013 available at http://www.statsghana.gov.gh/pop_stats.html

[21] Asante KP, Zandoh C, Dery DB, Brown C, Adjei G, Antwi-Dadzie Y et al.. Malaria epidemiology in the Ahafo area of Ghana. Malar. J. 2011. 10;211. Malaria j. 6;1:85 10.1186/1475-2875-10-211 PMC3171375 21801344

[22] Owusu-Agyei S, Ansong D, Asante K, Owusu SK, Owusu R, Brobby NAW (2009). Randomized controlled trial of RTS, S/AS02D and RTS, S/AS01E malaria candidate vaccines given according to different schedules in Ghanaian children. PLoS ONE.

[23] Owusu-Agyei S, Asante KP, Adjuik M, Adjei G, Awini E, Adams M (2009). Epidemiology of malaria in the forest-savanna transitional zone of Ghana. Malar J.

[24] GNHA 2002, National Health Accounts Ghana - World Health Organization accessed 3 May 2013 available on: www.who.int/entity/nha/country/gha/Ghana_NHA_report2002.pdf

[25] Asante KP, Abokyi L, Zandoh C, Owusu R, Awini E, Sulemana A (2010). Community perceptions of malaria and malaria treatment behaviour in a rural district of Ghana: implications for artemisinin combination therapy. BMC Public Health.

[26] Buabeng KO, Duwiejua M, Dodoo AN, Matowe LK, Enlund H (2007). Self-reported use of anti-malarial drugs and health facility management of malaria in Ghana.

[27] Ghana Health Service 2013,National Malaria Control Program.Strategies to achieve program objectives. http://www.ghanahealthservice.org/download/ghana_malaria_programme_review_final_report_june_2013.pdf. Accessed 3 Aug 2013.

[28] Dodoo NA, Fogg C, Asiimwe A, Nartey ET, Kodua A, Tenkorang O, et al. Pattern of drug utilization for treatment of uncomplicated malaria in urban Ghana following national treatment policy change to artemisinin-combination therapy. Malaria journal. 2009;8(1):1. 10.1186/1475-2875-8-2 PMC2647941 19123926

[29] Krech LA, Barlow CL, Siv L, Phanouvong S, Yuan WE, et al. Cambodian Ministry of Health Takes Decisive Actions in the Fight Against Substandard and Counterfeit Medicines. Tropical Medicine & Surgery. 2014;2:166. doi:10.4172/2329-9088.1000166.

[30] Osei-Safo D, Agbonon A, Konadu DY, Harrison JJEK, Edoh M, Gordon A. Evaluation of the quality of artemisinin-based antimalarial medicines distributed in Ghana and Togo. Malaria res treatment. 2014 10.1155/2014/806416 PMC4225840 25400975

[31] Aminake NM, Pradel G (2013). Antimalarial drugs resistance in Plasmodium falciparum and the current strategies to overcome them.

[32] W H O 2011b, WHO Global Malaria Programme, world malaria report 2011; ISBN 978 92 4 156440 3 (NLM Classification: WC 765)

[33] USAID 2009, Survey of the Quality of Selected Antimalarial Medicines Circulating in Madagascar, Senegal, and Uganda: U.S. PHARMACOPEIA. The Standard of Quality

[34] Caudron JM, Ford N, Henkens M, Macé C, Kiddle-Monroe R, Pinel J (2008). Substandard medicines in resource-poor settings: a problem that can no longer be ignored. Trop Med Int Health.

[35] Newton PN, Fernández FM, Plançon A, Mildenhall DC, Green MD, Ziyong L, et al. A Collaborative Epidemiological Investigation into the Criminal Fake Artesunate Trade in South East Asia. PLoS Med. 2008;5(2):32. doi:10.1371/journal.pmed.0050032. 10.1371/journal.pmed.0050032 PMC2235893 18271620

[36] Bate R, Hess K. The role of pre-shipment batch testing in ensuring good medicine quality. Malaria World J. 2012;3:11. 10.5281/zenodo.10997689 PMC11153354 38854887

[37] Binagwaho A, Bate R, Gasana M, Karema C, Mucyo Y, Mwesigye JP (2013). Combatting Substandard and Falsified Medicines: A View from Rwanda. PLoS Med.

[38] Nugent R, Back E, Beith A. The race against drug resistance. Washington DC: Center for Global Development; 2010.

[39] Asante KP, Owusu R, Dosoo D, Awini E, Adjei G, Amenga Etego S, Chandramohan D, Owusu-Agyei S. Adherence to Artesunate-Amodiaquine Therapy forUncomplicated Malaria in Rural Ghana: A Randomised Trial of Supervised versus Unsupervised DrugAdministration. Journal of tropical medicine. 2009;529583. ISSN 1687-9686. doi:10.1155/2009/529583. 10.1155/2009/529583 PMC2836893 20339565

[40] Ghana National Drug Policy (GNDP). Ministry of Health (MoH), Republic of Ghana. Second Edition, July 2004.

[41] Ministry of Health, 2004: Standards Treatment Guidelines - Ghana, Ghana National Drug Programme

[42] Ministry of Health 2009, Anti-malaria Drug Policy for Ghana, accessed 10 June 2013, apps.who.int › Medicines Policy

[43] Nsimba EDS (2008). Problems Associated With Substandard and Counterfeit Drugs in Developing Countries: A Review Article on Global Implications of Counterfeit Drugs in the Era of Anti-Retroviral (ARVS) Drugs in A Free Market Economy. East African j Public Health.

[44] El-Duah M, Ofori-Kwakye K (2012). Substandard artemisinin-based antimalarial medicines in licensed retail pharmaceutical outlets in Ghana. J Vector Borne Dis.

[45] Gopakumar KM 2013, WHO: Members agreement on list of behaviours linked to compromised medical products, TWN Info Service on Health Issues. Third World Network accessed 30 July 2013. http://www.twn.my/title2/intellectual_property/info.service/2013/ipr.info.130708.html

